# Essential Assembly Factor Rpf2 Forms Novel Interactions within the 5S RNP in *Trypanosoma brucei*

**DOI:** 10.1128/mSphere.00394-17

**Published:** 2017-10-18

**Authors:** Anyango D. Kamina, Daniel Jaremko, Linda Christen, Noreen Williams

**Affiliations:** Department of Microbiology and Immunology and Witebsky Center for Microbial Pathogenesis and Immunology, University at Buffalo, Buffalo, New York, USA; Carnegie Mellon University

**Keywords:** RNA interference, biochemistry, cell biology, protein-RNA interactions, protein-protein interactions

## Abstract

Ribosome biogenesis is a highly complex and conserved cellular process that is responsible for making ribosomes. During this process, there are several assembly steps that function as regulators to ensure proper ribosome formation. One of these steps is the assembly of the 5S ribonucleoprotein particle (5S RNP) in the central protuberance of the 60S ribosomal subunit. In eukaryotes, the 5S RNP is composed of 5S rRNA, ribosomal proteins L5 and L11, and assembly factors Rpf2 and Rrs1. Our laboratory previously showed that in *Trypanosoma brucei*, the 5S RNP is composed of 5S rRNA, L5, and trypanosome-specific RNA binding proteins P34 and P37. In this study, we characterize an additional component of the 5S RNP, the *T. brucei* homolog of Rpf2. This is the first study to functionally characterize interactions mediated by Rpf2 in an organism other than fungi. *T*. *brucei* Rpf2 (TbRpf2) was identified from tandem affinity purification using extracts prepared from protein A-tobacco etch virus (TEV)-protein C (PTP)-tagged L5, P34, and P37 cell lines, followed by mass spectrometry analysis. We characterized the binding interactions between TbRpf2 and the previously characterized members of the *T. brucei* 5S RNP. Our studies show that TbRpf2 mediates conserved binding interactions with 5S rRNA and L5 and that TbRpf2 also interacts with trypanosome-specific proteins P34 and P37. We performed RNA interference (RNAi) knockdown of TbRpf2 and showed that this protein is essential for the survival of the parasites and is critical for proper ribosome formation. These studies provide new insights into a critical checkpoint in the ribosome biogenesis pathway in *T. brucei*.

**IMPORTANCE** *Trypanosoma brucei* is the parasitic protozoan that causes African sleeping sickness. Ribosome assembly is essential for the survival of this parasite through the different host environments it encounters during its life cycle. The assembly of the 5S ribonucleoprotein particle (5S RNP) functions as one of the regulatory checkpoints during ribosome biogenesis. We have previously characterized the 5S RNP in *T. brucei* and showed that trypanosome-specific proteins P34 and P37 are part of this complex. In this study, we characterize for the first time the interactions of the homolog of the assembly factor Rpf2 with members of the 5S RNP in another organism besides fungi. Our studies show that Rpf2 is essential in *T. brucei* and that it forms unique interactions within the 5S RNP, particularly with P34 and P37. These studies have identified parasite-specific interactions that can potentially function as new therapeutic targets against sleeping sickness.

## Abstract (precis)

*Trypanosoma brucei* is the parasitic protozoan that causes African sleeping sickness. Ribosome assembly is essential for the survival of this parasite through the different host environments it encounters during its life cycle. The assembly of the 5S ribonucleoprotein particle (5S RNP) functions as one of the regulatory checkpoints during ribosome biogenesis. We have previously characterized the 5S RNP in *T. brucei* and showed that trypanosome-specific proteins P34 and P37 are part of this complex. In this study, we characterize for the first time the interactions of the homolog of the assembly factor Rpf2 with members of the 5S RNP in another organism besides fungi. Our studies show that Rpf2 is essential in *T. brucei* and that it forms unique interactions within the 5S RNP, particularly with P34 and P37. These studies have identified parasite-specific interactions that can potentially function as new therapeutic targets against sleeping sickness.

## INTRODUCTION

*Trypanosoma brucei* parasites are the causative agents of African trypanosomiasis both in humans (sleeping sickness) and in animals (nagana) (1). These parasites have complex life cycles that take place within both a mammalian host and an insect vector, the tsetse fly (2). During its life cycle, *T. brucei* undergoes several morphological and biochemical changes. Its rate of protein synthesis also changes depending on whether the parasites are replicating or not and in order to adapt to the different host environments it encounters (3–5).

Ribosome biogenesis is the essential process of making ribosomes, the cellular machines responsible for protein synthesis (6, 7). In eukaryotes, ribosome biogenesis begins in the nucleolus with the transcription of a 35S rRNA precursor by RNA polymerase I (8). This precursor is processed into three of the four rRNA species (18S, 5.8S, and 25S) and joins together with ribosomal proteins and accessory factors to form the 60S and 40S preribosomal subunits (6, 9). These two subunits mature separately in the nucleus and are exported to the cytoplasm where they join to form the 80S ribosome. In most eukaryotes, the fourth rRNA, 5S rRNA, is transcribed in the nucleoplasm by RNA polymerase III. Following transcription, processing, and maturation, 5S rRNA forms the 5S ribonucleoprotein particle (5S RNP) with ribosomal protein L5 and is trafficked to the nucleolus (10). In the nucleolus, additional factors, ribosomal protein L11 and two assembly factors, ribosome production factor 2 (Rpf2) and regulator of ribosome synthesis 1 (Rrs1), are added to the 5S RNP (11–13). Assembly of the 5S RNP into the 60S preribosomal subunit is important for its maturation and nuclear export (11). In *Saccharomyces cerevisiae*, the absence of any one of the 5S RNP components leads to the lack of recruitment of the other members of the 5S RNP to the 60S preribosomal subunit and an accumulation of immature 60S preribosomal subunits within the nucleus (11). Also in *S. cerevisiae*, the absence of proper assembly of the 5S RNP inhibits the final processing steps involved in forming mature 25S rRNA (11, 14).

Our laboratory previously showed that in *T. brucei* the 5S RNP is composed of 5S rRNA, L5, and trypanosome-specific RNA binding proteins P34 and P37 (15, 16). We also showed that P34 and P37 are essential proteins and are involved in several stages of the 60S ribosomal subunit biogenesis (17, 18). Although P37 has an 18-amino-acid N-terminal extension compared to P34, these two proteins are structurally and functionally similar. To determine the complexity of the 5S RNP in *T. brucei*, we performed tandem affinity purification using protein A-tobacco etch virus (TEV)-protein C (PTP)-tagged L5, P34, and P37 proteins. From these studies, we identified the *T. brucei* homolog of the Rpf2 protein as well as the homologs of ribosomal protein L11 and Rrs1. In this study, we characterize *T. brucei* Rpf2 (TbRpf2) to determine whether it is an essential protein and also to identify the conserved and unique features of this protein in *T. brucei*.

## RESULTS

### Identification of TbRpf2.

Our laboratory previously showed that the *T. brucei* 5S RNP is composed of 5S rRNA, L5, and trypanosome-specific proteins P34 and P37. In order to identify any additional components of the 5S RNP, we tagged the known protein members using protein A-TEV-protein C (PTP) tag generating three *T. brucei* cell lines expressing PTP-L5-, PTP-P34-, and PTP-P37-tagged proteins. We performed tandem affinity purification using extracts prepared from all three cell lines and had them analyzed by mass spectrometry (Thermo Scientific LTQ Orbitrap Elite, Fred Hutchinson Cancer Center, Seattle, WA) to identify associating proteins. All of the identified associating proteins are listed in Tables S1 to S3 in the supplemental material.

10.1128/mSphere.00394-17.4 TABLE S1  Proteins identified from mass spectrometry analysis of PTP-L5 purification. Download TABLE S1, PDF file, 0.1 MB. Copyright © 2017 Kamina et al. 2017 Kamina et al. This content is distributed under the terms of the Creative Commons Attribution 4.0 International license.

10.1128/mSphere.00394-17.5 TABLE S2  Proteins identified from mass spectrometry analysis of PTP-P34 purification. Download TABLE S2, PDF file, 0.1 MB. Copyright © 2017 Kamina et al. 2017 Kamina et al. This content is distributed under the terms of the Creative Commons Attribution 4.0 International license.

10.1128/mSphere.00394-17.6 TABLE S3  Proteins identified from mass spectrometry analysis of PTP-P37 purification. Download TABLE S3, PDF file, 0.1 MB. Copyright © 2017 Kamina et al. 2017 Kamina et al. This content is distributed under the terms of the Creative Commons Attribution 4.0 International license.

**FIG 1:**
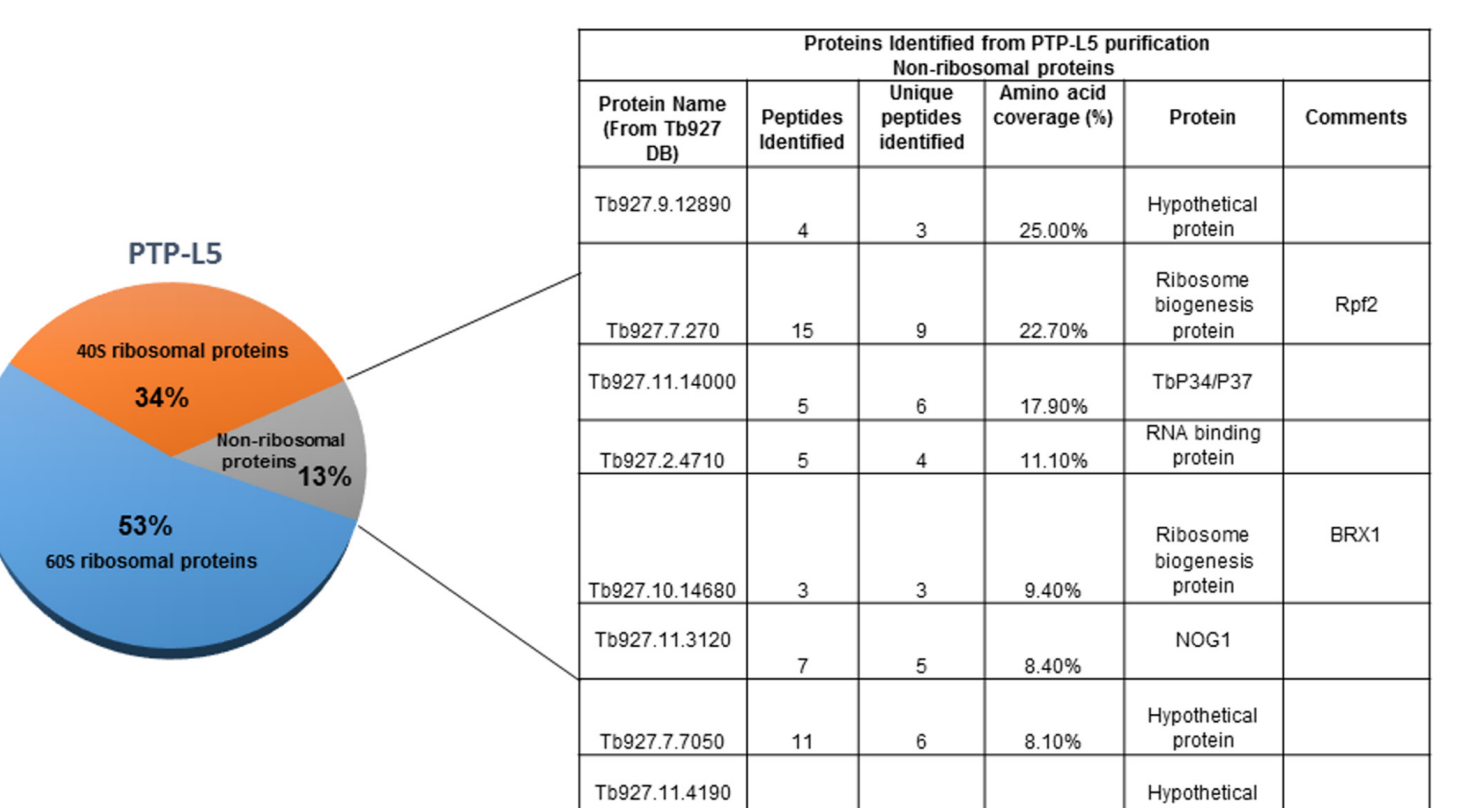
Summary of proteins identified from mass spectrometry analysis of PTP-L5 purification. In the table to the right of the pie chart, the Protein Name column shows the names of the sequences from the protein database. The Peptides Identified column shows the number of filtered peptides in the run that were matched to this sequence. The Unique peptides identified column shows the numbers of unique filtered peptides in the run that were matched to this sequence. Amino acid coverage shows the percentage of the amino acid sequence covered by the unique peptides. The Comments column shows the best name/description for the identified protein.

We then sorted the identified proteins. First, all of the contaminating proteins (e.g., keratin), proteins with less than two unique peptides identified, and hypothetical proteins that did not have a reported function in the trypanosome database were removed from the list (Fig. 1 and Fig. S1 and S2). Next, all of the homologs to 60S and 40S ribosomal proteins were grouped separately (Fig. 1 and Fig. S1 and S2). Finally, nonribosomal proteins and hypothetical proteins that had a reported function in the trypanosome database were grouped together (Fig. 1 and Fig. S1 and S2).

10.1128/mSphere.00394-17.1 FIG S1  Summary of proteins identified from mass spectrometry analysis of PTP-P34 purification. In the table to the right of the pie chart, the Protein Name column shows the names of the sequences from the protein database. The Peptides Identified column shows the number of filtered peptides in the run that were matched to this sequence. The Unique peptides identified column shows the numbers of unique filtered peptides in the run that were matched to this sequence. Amino acid coverage shows the percentage of the amino acid sequence covered by the unique peptides. The Comments column shows the best name/description for the identified protein. Download FIG S1, PDF file, 0.6 MB. Copyright © 2017 Kamina et al. 2017 Kamina et al. This content is distributed under the terms of the Creative Commons Attribution 4.0 International license.

10.1128/mSphere.00394-17.2 FIG S2  Summary of proteins identified from mass spectrometry analysis of PTP-P37 purification. In the table to the right of the pie chart, the Protein Name column shows the names of the sequences from the protein database. The Peptides Identified column shows the number of filtered peptides in the run that were matched to this sequence. The Unique peptides identified column shows the numbers of unique filtered peptides in the run that were matched to this sequence. Amino acid coverage shows the percentage of the amino acid sequence covered by the unique peptides. The Comments column shows the best name/description for the identified protein. Download FIG S2, PDF file, 0.5 MB. Copyright © 2017 Kamina et al. 2017 Kamina et al. This content is distributed under the terms of the Creative Commons Attribution 4.0 International license.

Our first priority was to identify and characterize associating proteins that are part of the 5S RNP. We identified the *T. brucei* homolog of the *S. cerevisiae* Rpf2 protein (Tb927.7.270) and found that it associates with L5 and with P37 (nonribosomal proteins in Fig. 1 and in Tables S1 and S3). We also identified the *T. brucei* homolog of L11, which associates with L5, P34, and P37 (Tables S1 to S3). The homolog of Rrs1 was identified as associating with L5; however, it was not included in our sorted data, because less than two unique peptides were identified, which was below our cutoff. In this study, we characterize TbRpf2.

**FIG 2:**
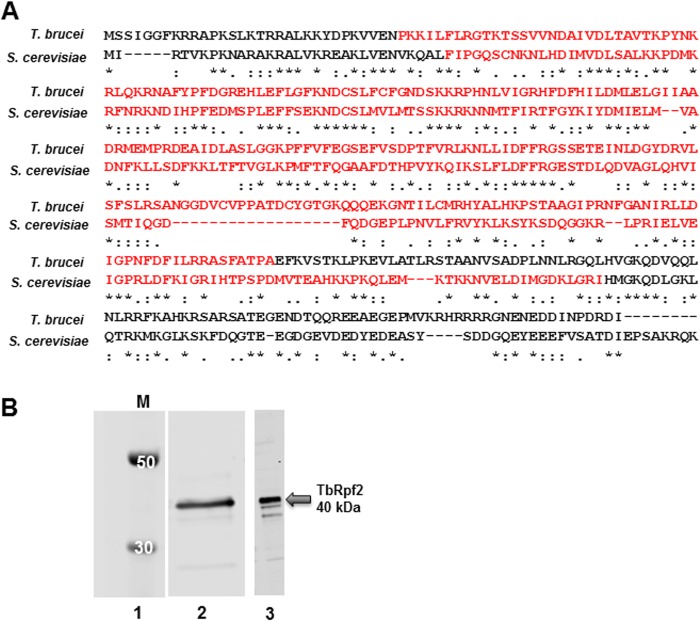
(A) Sequence alignment between *T. brucei* Rpf2 protein and *S. cerevisiae* Rpf2 protein using Tcoffee. Amino acids in the Brix domain are shown in red. Amino acids that are identical in the two proteins (*), amino acids in the same group (:), and amino acids that are different (.) are indicated below the sequence alignment. (B) Western blot analysis of cell extracts prepared from *T. brucei* 427 procyclic cells (lane 2) and recombinant TbRpf2 protein (lane 3). Lane 1 contains molecular mass markers (in kilodaltons). The Western blot was performed using an anti-Rpf2 peptide antibody. All Western blot analyses were performed on three biological replicates. and representative blots are shown.

Next we performed sequence alignment between TbRpf2 and Rpf2 from other trypanosomatids and determined that the sequences were similar (76% similar to *Trypanosoma cruzi* Rpf2 and 63% similar to *Leishmania donovani* Rpf2). We then performed sequence alignments between TbRpf2 and *S*. *cerevisiae* Rpf2 (ScRpf2) and showed that these two proteins share 36% sequence similarity. Rpf2 is a member of the biogenesis of ribosomes in *Xenopus* (BRIX) family of proteins, which are characterized to function in the early stages of ribosome biogenesis (19). The common feature of these proteins is the presence of a BRIX domain, which is mainly composed of charged residues with a proposed function of mediating RNA binding (19, 20). To further characterize the features of the TbRpf2 protein, we compared the sequences of the BRIX domains of TbRpf2 and ScRpf2. Our sequence alignment showed that the sequence identity between the TbRpf2 and ScRpf2 BRIX domain is 32% (Fig. 2A, red residues).

Structural studies showed that Rpf2 and Rrs1 form a heterodimer and that Rpf2 is unstable when expressed alone (12). In order to determine whether this phenotype is conserved in *T. brucei*, we expressed recombinant TbRpf2 in *E. coli* without coexpression with Rrs1. We also prepared whole-cell extracts (WCE) from *T. brucei* cells and performed Western blot analysis using an anti-TbRpf2 peptide antibody. The TbRpf2 protein migrated at its proposed molecular mass of 40 kDa in WCE (Fig. 2B, lane 2) and as a recombinant protein (Fig. 2B, lane 3). Our results show that TbRpf2 can be expressed as a soluble protein without Rrs1.

### TbRpf2 interacts with *T*. *brucei* 5S rRNA.

**FIG 3:**
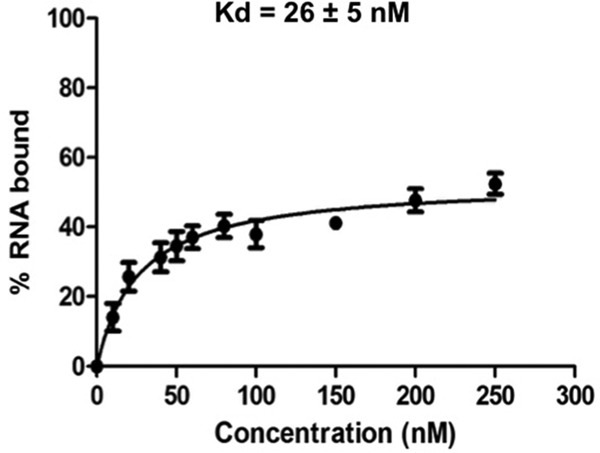
TbRpf2 binds to 5S rRNA. Recombinant TbRpf2 was incubated with radiolabeled 5S rRNA, and their interactions were analyzed using filter binding assays. The values obtained from the quantification of the bound and free 5S rRNA [bound/(bound + free)] was used to calculate the binding affinity (*K*_*d*_) of 5S rRNA for the TbRpf2 protein. The experiments were performed in biological triplicates, and the error bars indicate the standard errors of means (SEM) from the three biological replicates.

Studies of the protein-RNA interactions within the 5S RNP in fungi showed that Rpf2 associates with 5S rRNA (11, 21). Therefore, we characterized the interaction between TbRpf2 and 5S rRNA in order to determine whether these interactions are conserved in *T. brucei*. We expressed recombinant TbRpf2 in *E. coli* and used it in filter binding assays with radiolabeled 5S rRNA. The results showed that TbRpf2 bound 5S rRNA, and the calculated binding affinity (*K*_*d*_) was 26 ± 5 nM (Fig. 3). These results show that recombinant TbRpf2 protein is functional in the absence of Rrs1 and is able to bind to 5S rRNA.

### TbRpf2 interacts with *T. brucei* L5 protein.

**FIG 4:**
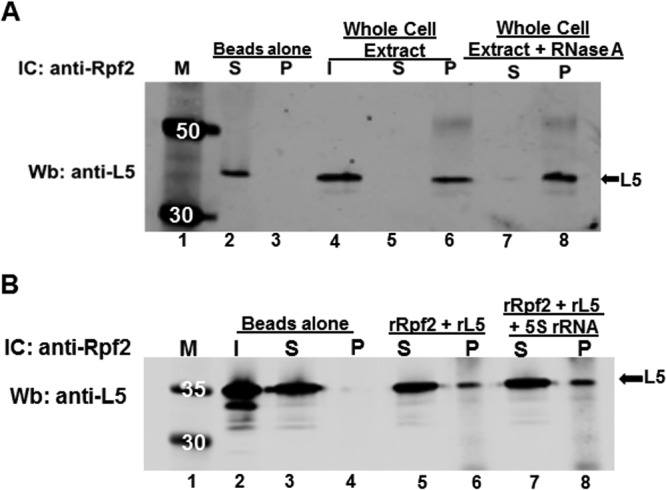
Rpf2 binds to L5 *in vivo* and *in vitro*. (A) Interaction between TbRpf2 and L5 from whole-cell extracts (WCE) (100 μg) with and without RNase A treatment. (B) Interaction between recombinant TbRpf2 (rRpf2) and recombinant L5 (rL5) protein (1 μg each) with and without the presence of 5S rRNA (1 μg). The proteins were incubated with beads cross-linked with anti-Rpf2 antibody to capture Rpf2 with L5 bound to it (L5 at 36 kDa). The immune captured (IC) complexes were analyzed by Western blotting (Wb) using anti-L5 antibody. No antibody was used in the Beads alone lanes. Lanes: I, input (L5 antibody control, 10% [WCE] or 50% [recombinant protein] of total protein used in the reaction]); S, supernatant (unbound L5); P, pellet (bound L5); M, molecular mass markers (in kilodaltons).

Several studies have shown that Rpf2 binds to L5 protein (11, 13). Therefore, we characterized whether the interaction between TbRpf2 and L5 is conserved in *T. brucei*. First, we examined the interaction *in vivo* using WCE prepared from *T. brucei* procyclic cells. We performed the immune capture using anti-Rpf2 antibody and Western blot analysis using anti-L5 antibody. We showed that TbRpf2 and L5 clearly associate (Fig. 4A, lane 6). To determine whether the *in vivo* association was due to RNA facilitating the interaction between the two proteins, we treated the WCE with RNase A to cleave any accessible RNA. Surprisingly, results showed that there was an increase in the amount of L5 that associated with TbRpf2 in the RNase A-treated extracts (Fig. 4A, compare lane 6 and lane 8). This result suggests that the presence of RNAs could be hindering direct binding between L5 and TbRpf2 *in vivo*. Next we prepared recombinant TbRpf2 and L5 proteins and characterized their *in vitro* interaction using immune capture assays. The results showed that TbRpf2 and L5 directly interact *in vitro* (Fig. 4B, lane 6). Since we observed that depletion of RNAs increased the interaction between TbRpf2 and L5 *in vivo*, we wanted to determine whether it was 5S rRNA that influenced this protein-protein interaction. We incubated TbRpf2, L5, and 5S rRNA *in vitro* and then analyzed the interaction in an immune capture assay. The results showed that the addition of 5S rRNA does not have a significant effect on the interaction between TbRpf2 and L5 (Fig. 4B, compare lane 6 to lane 8). Overall, our results show that TbRpf2 interacts with L5 both *in vivo* and *in vitro* and that 5S rRNA does not have an effect on this interaction.

### TbRpf2 interacts with trypanosome-specific proteins P34 and P37.

**FIG 5:**
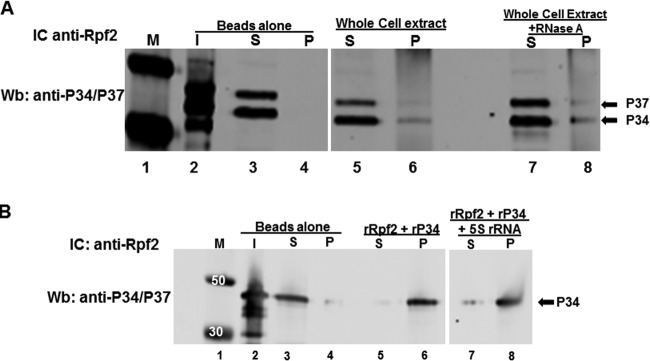
Rpf2 binds to P34 *in vivo* and *in vitro*. (A) Interaction between TbRpf2 and P34, P37 from whole-cell extracts (WCE) (100 μg) with and without RNase A treatment. (B) Interaction between recombinant TbRpf2 (rRpf2) (1 μg) and recombinant P34 (rP34) (1 μg) protein with and without 5S rRNA (1 μg). The proteins were incubated with beads cross-linked with anti-Rpf2 antibody to capture TbRpf2 with P34 bound to it. The immune captured (IC) complexes were analyzed by Western blotting (Wb) using anti-P34/P37 antibody (P34 migrates to 34 kDa, and P37 migrates to 37 kDa). No antibody was used in the Beads alone lanes. Lanes: I, input (P34 antibody control, 10% [WCE] or 50% [recombinant protein] of total protein used in the reaction); S, supernatant (unbound P34); P, pellet (bound P34); M, molecular mass markers (in kilodaltons).

In addition to the conserved members, 5S rRNA and L5, the *T. brucei* 5S RNP is also composed of trypanosome-specific RNA binding proteins P34 and P37. These proteins interact with 5S rRNA and L5 both *in vivo* and *in vitro* and are important for the stability of 5S rRNA and for export of the 60S subunit to the cytoplasm (15–17). From our PTP pulldown assays, we identified TbRpf2 as an associating protein with P37 but not with P34. Therefore, we performed immune capture assays to further characterize these interactions *in vivo* and *in vitro*. We prepared WCE from *T. brucei* procyclic cells and showed that TbRpf2 associates with both P34 and P37 *in vivo* (Fig. 5A, lane 6). We next wanted to determine whether the *in vivo* association of TbRpf2 with P34 and P37 is affected by the presence of RNAs. Thus, we treated the WCE with RNase A to eliminate any RNA that is accessible to degradation and then performed immune capture experiments. The results showed that RNase A treatment did not have an effect on the association of TbRpf2 with P34 and P37 (Fig. 5A, compare lane 6 and lane 8).

Since our laboratory showed that P34 and P37 are functionally similar, we used recombinant P34 protein in *in vitro* immune capture experiments with recombinant TbRpf2. Interestingly, these results showed that P34 and TbRpf2 directly bind to each other even though we did not identify TbRpf2 as an associating protein from the PTP-P34 pulldown (Fig. 5B, lane 6). Although we showed that RNAs had no effect on the interaction between TbRpf2 and P34 and P37 *in vivo*, we wanted to determine whether 5S rRNA had an effect on the *in vitro* interactions between TbRpf2 and P34. This is because previous studies in our laboratory showed that 5S rRNA enhances the interaction between L5 and trypanosome-specific protein P34 (15). Our results showed that the addition of 5S rRNA does not have a significant effect on this protein-protein interaction (Fig. 5B, compare lane 6 to lane 8). These results show that TbRpf2 interacts with P34 and P37 and that RNAs do not affect this interaction.

### TbRpf2 is an essential protein.

**FIG 6:**
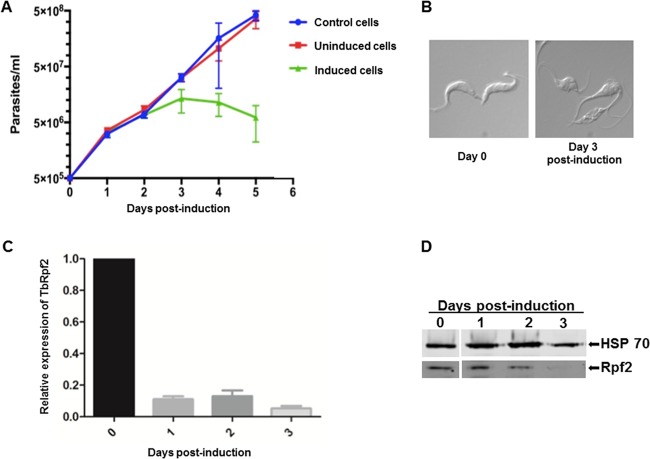
TbRpf2 is an essential protein in *T. brucei*. (A) Growth curves comparing the cell growth of wild-type cells (blue line), transfected but uninduced cells (red line), and transfected induced cells (green line). (B) Differential imaging contrast (DIC) images of uninduced cells (day 0) and RNAi-induced cells (day 3). (C) qPCR was used to analyze the extent of the Rpf2 RNAi-induced knockdown. The values of expression for each day were compared to the value at day 0. The value of 1 corresponds to day 0 transcript levels, and values below 1 indicate transcript level depletion. The experiments were performed in biological triplicates, and the error bars indicate standard errors from the three experiments. (D) Western blot analysis of the protein levels of TbRpf2 on days 0, 1, 2, and 3 postinduction. HSP 70 is used as a loading control.

We next generated an Rpf2 RNA interference (RNAi) cell line to determine whether TbRpf2 is an essential protein in *T. brucei*. We analyzed the growth of the wild-type and TbRpf2 RNAi cells in the presence and absence of tetracycline (Fig. 6A). Comparison of the growth of untransfected wild-type cells (Fig. 6A, blue line) to that of transfected but uninduced cells (Fig. 6A, red line) showed that the cells grew similarly. This shows that the TbRpf2 RNAi construct had no negative effect on cell growth. Upon induction of TbRpf2 RNAi knockdown, the parasites underwent an arrest in cell growth by day 3 postinduction (Fig. 6A, green line) and began to die. We examined the cells using differential imaging contrast (DIC) to observe the effect that induction of TbRpf2 knockdown had on cellular morphology. The results showed that by day 3 postinduction, the cells were no longer able to fully divide (Fig. 6B). We performed quantitative PCR (qPCR) and Western blot analysis using anti-Rpf2 peptide antibody to confirm that the observed growth phenotype was due to knockdown of the TbRpf2 transcript and protein. qPCR results showed that the levels of TbRpf2 mRNA decreased to 80% compared to day 0 levels and that the protein levels decreased by 50% by day 2 postinduction (Fig. 6C and D). These decreases were observed further on day 3 postinduction when the TbRpf2 mRNA decreased to 90% compared to day 0 levels and the protein was depleted (Fig. 6C and D). Taken together, these results show that TbRpf2 is an essential protein in *T. brucei*.

### Loss of TbRpf2 protein leads to a small decrease in the protein levels of the other members of the 5S RNP.

**FIG 7:**
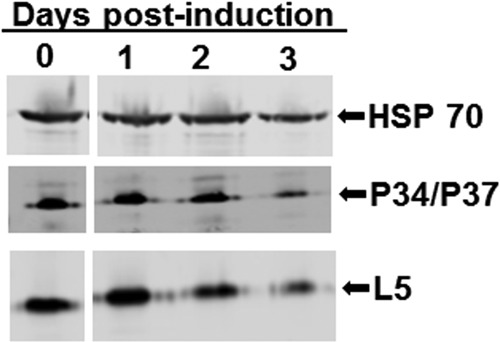
Loss of TbRpf2 leads to a small decrease in the protein levels of the other members of the 5S RNP. Cell extracts were prepared from Rpf2 RNAi cells collected on days 0, 1, 2, and 3 postinduction and used in Western blotting using anti-P34/P37 and anti-L5 antibodies. HSP 70 was used as a loading control.

To determine whether the steady-state levels of the protein components of the 5S RNP are altered upon loss of TbRpf2, cell extracts from days 0, 1, 2, and 3 postinduction of TbRpf2 RNAi knockdown were analyzed. We performed Western blotting to analyze the protein levels of P34, P37, L5, and HSP 70, which was used as a loading control. We then quantified the signal for each day in order to determine whether there was a decrease in the protein levels after RNAi induction. We normalized the values obtained from these quantifications to the value obtained on day 0, which was set at 100%. The results showed that by day 2 postinduction, when TbRpf2 protein level is reduced by approximately 50%, there was an approximately 15% decrease in the levels of P34 and P37 proteins and a 10% decrease in the levels of L5 protein (Fig. 7, day 2 postinduction). By day 3 postinduction, there was an 80% decrease in the levels of P34 and P37 and a 70% decrease in the levels of L5 (Fig. 7, day 3 postinduction). These results show that knockdown of TbRpf2 has a small effect on the protein levels of P34, P37, and L5.

### Loss of TbRpf2 leads to a defect in ribosome biogenesis.

**FIG 8:**
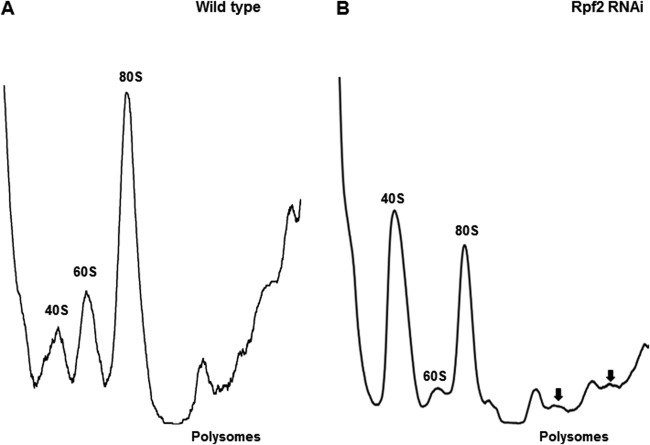
Loss of TbRpf2 leads to defects in ribosome assembly. Polysome extracts were prepared from wild-type cells (A) and Rpf2 RNAi cells (B) and then sedimented on 10 to 40% sucrose gradients. The positions of half-mers are indicated by the black arrows.

In *S. cerevisiae*, depletion of Rpf2 leads to aberrant export of premature 60S preribosomal subunits to the nucleoplasm that cannot be exported to the cytoplasm, an accumulation of the 40S ribosomal subunit, a decrease in ribosome formation, and a defect in the final processing step needed to form 25S rRNA (11, 14). To determine whether knockdown of Rpf2 in *T. brucei* showed a similar effect, we prepared cell extracts from uninduced and RNAi-induced cells. These extracts were separated on sucrose gradients, and then their sedimentation profiles were analyzed by monitoring the UV absorbance at 260 nm. The wild-type profile shows the positions of the 40S and 60S ribosomal subunits, 80S ribosome, and polysomes (Fig. 8A). We compared this profile to the profile obtained from extracts prepared from Rpf2 RNAi-induced cells collected on day 2 postinduction. We observed that knockdown of TbRpf2 leads to an increase in the 40S ribosomal subunit, a decrease in the 60S ribosomal subunit, and a decrease in the 80S ribosome and polysomes (Fig. 8B). These results suggest that loss of TbRpf2 leads to a defect in formation of functional 60S ribosomal subunits. Thus, fewer 60S ribosomal subunits are able to join with the 40S ribosomal subunits to form the 80S ribosome and polysomes. This hypothesis is further supported by our observation of the formation of half-mers (incomplete polysomes) along with some polysomes (Fig. 8B, black arrows). This phenotype is similar to that observed in *S. cerevisiae* and suggests that *T. brucei* Rpf2 protein has a conserved function in the biogenesis of the 60S ribosomal subunit.

## DISCUSSION

Our understanding of the function and assembly of the 5S RNP is almost completely based on studies of fungi, particularly *S. cerevisiae*. These studies showed that the assembly of the 5S RNP is important for biogenesis of the 60S ribosomal subunit and for proper processing of the rRNA precursors that will form mature 25S rRNA (11). All of the protein members of this complex, including Rpf2, Rrs1, L5, and L11, are essential for cell growth, and depletion of any of these components leads to formation of a 60S subunit that cannot be exported to the cytoplasm for further maturation (11, 14, 22). Studies of this complex in *S. cerevisiae* showed that they form protein-RNA and protein-protein interactions *in vivo* and *in vitro* and that Rpf2 and Rrs1 form a strong interaction with each other compared to their interaction with the other members of the complex (11). Structural studies showed that only when Rpf2 and Rrs1 were coexpressed together were they able to form a crystal structure. These crystal structures revealed that Rpf2 and Rrs1 form and function as a heterodimer where Rrs1 completes the C-terminal BRIX domain of Rpf2 (12, 13, 21). Since the function and assembly of the 5S RNP has been extensively studied in only one organism, we were interested in characterizing the degree of conservation of this entire complex in *T. brucei*. Since *T. brucei* is an early diverging protozoan, characterization of this essential complex in this organism will elucidate the unique and conserved components of the 5S RNP.

Our laboratory already showed that in *T. brucei* the 5S RNP is composed of 5S rRNA, L5, and trypanosome-specific RNA binding proteins P34 and P37. To identify additional members of the *T. brucei* 5S RNP, we tagged the known protein components (L5, P34, and P37) with an PTP tag. This allowed us to perform tandem affinity purification followed by mass spectrometry analysis to identify associating proteins. We identified many ribosomal (40S and 60S proteins), nonribosomal, and hypothetical proteins associating with L5, P34, and P37 (Fig. 1, Fig. S1 and S2, and Tables S1 to S3). More of the ribosomal proteins associating with the 5S RNP protein members were 60S ribosomal proteins as anticipated, since this complex associates with the 60S biogenesis pathway. From these analyses, we identified *T. brucei* homologs of the 5S RNP components, L11, Rpf2, and Rrs1. In this study, we characterized the TbRpf2 protein (Fig. 2).

As previously mentioned, studies showed that Rpf2 binds directly to 5S rRNA and L5 both *in vivo* and *in vitro* (11, 13). Therefore, we first characterized the protein-RNA and protein-protein interactions within the 5S RNP to determine which interactions are conserved and which are unique in *T. brucei*. In our studies, we were able to express a soluble and functional recombinant TbRpf2 protein without Rrs1 (Fig. 2B, lane 3). We used this recombinant TbRpf2 protein in filter binding assays to characterize its interaction with 5S rRNA. We calculated the binding affinity of 5S RNA for TbRpf2 to be 26 ± 5 nM (Fig. 3). Most of the *in vitro* studies of the interaction between Rpf2 and 5S rRNA have been performed using the *S. cerevisiae* Rpf2-Rrs1 dimer (12, 13). The binding affinity of 5S rRNA for the ScRpf2-Rrs1 heterodimer was calculated to be 59 nM. These studies also showed that only Rpf2 mediates binding to 5S rRNA, and significantly, that when Rpf2 was expressed without Rrs1, it was unstable (12). These findings suggest that in fungi Rrs1 may be important in improving the functional ability of Rpf2 but not in direct binding (12, 13, 21). The *in vitro* interaction between Rpf2 and 5S rRNA, where Rpf2 was expressed alone, has been characterized only in *Chaetomium thermophilum*, a filamentous fungus that is stable at high temperatures, which suggests that there are features of Rpf2 that are unique in this fungal species (13). Due to the unique nature of TbRpf2, we have quantified for the first time the *in vitro* binding affinity of 5S rRNA to TbRpf2 protein alone and showed that the affinity is stronger in *T. brucei* for TbRpf2 alone than for the ScRpf2-Rrs1 heterodimer. These results suggest that there are unique features about the TbRpf2 protein that allows it to be stable and have a stronger affinity for 5S rRNA when expressed alone. Similar to *C. thermophilum*, the increased stability of TbRpf2 may contribute to the ability of *T. brucei* to survive a range of different temperatures (27°C to 37°C) it encounters during its life cycle. Ongoing studies in our laboratory will determine how the coexpression of Rpf2 and Rrs1 as a heterodimer influences the characterized interaction between TbRpf2 and 5S rRNA.

Next we analyzed the interaction between TbRpf2 and L5 using immune capture assays and showed that they interact both *in vivo* and *in vitro* (Fig. 4). It was interesting that when we treated our extracts with RNase A in order to degrade any RNA that was associated in the extract, we observed an increase in the association of L5 with TbRpf2 *in vivo* (Fig. 4A, lane 8). This suggests that there could be RNAs that occlude strong binding between TbRpf2 and L5. From this result, we wanted to determine whether it was 5S rRNA that occluded this binding between L5 and TbRpf2. However, when we added 5S rRNA to the *in vitro* interaction between TbRpf2 and L5, our results showed that this RNA does not have a significant effect on their interaction. In *S. cerevisiae*, one of the models for the order of assembly of the 5S RNP to the 60S subunit suggests that L5 and L11 are translocated by symportin 1 (Syo1) chaperone protein to the nucleolus where they bind to 5S rRNA. This complex is then assembled by the Rpf2-Rrs1 heterodimer into the 60S subunit where they interact with 25S rRNA (23). We showed that in *T. brucei* 5S rRNA and L5 interact in the nucleoplasm prior to assembly to the 60S subunit. We do not know when in the assembly process 5S rRNA and L5 interact with TbRpf2. Additionally, the homolog of Syo1 has not been identified in *T. brucei*. From our results, we hypothesize that the order of assembly is similar to the model described for *S. cerevisiae*. Thus, the effect of RNA on the interaction between TbRpf2 and L5 *in vivo* could be due to their interaction with 25S rRNA once 5S rRNA and L5 make contact with the 60S subunit. Prior to this study, the direct interaction between Rpf2 and L5 has been reported only in one study in *S. cerevisiae* and in one study in *C. thermophilum*, where the authors showed that Rpf2 binds to L5 both *in vivo* and *in vitro* (11, 13). However, these studies did not determine whether RNA influenced the interaction between TbRpf2 and L5. Our studies show that the interaction between Rpf2 and L5 protein is conserved in *T. brucei*, and we also show for the first time that RNA influences this interaction.

We also used immune capture assays to characterize the interaction between TbRpf2 and trypanosome-specific proteins P34 and P37. Interestingly, we determined that TbRpf2 associates with P34 and P37 *in vivo* (Fig. 5A). These results further support our PTP-P37 pulldown data that showed that TbRpf2 associates with P37. Our results also show that TbRpf2 associates with P34 even though we did not observe this association in our PTP-P34 pulldown analysis. However, it could be that TbRpf2 was not identified as an associating protein with P34 from the mass spectrometry analysis due to differences in the depth of peptide coverage between the PTP-P34 and PTP-P37 analysis. Unlike our studies with L5, when we treated the whole-cell extracts with RNase A to degrade accessible RNAs, there was no effect on the interaction between TbRpf2, P34, and P37. This underscores differences in the binding interactions of TbRpf2 with other protein components of the *T. brucei* 5S RNP *in vivo*. Using recombinant proteins, we demonstrated that TbRpf2 binds directly to P34 *in vitro* (Fig. 5B) and that the addition of 5S rRNA does not significantly impact the interaction (Fig. 5B). These results further support our hypothesis on the order of assembly of the *T. brucei* 5S RNP. Similar to previous studies with L5, our laboratory showed that P34 and P37 associate with L5 and 5S rRNA in the nucleoplasm prior to assembly to the 60S subunit (18). This suggests that 5S rRNA, L5, P34, and P37 associate with each other first and then interact with the Rpf2-Rrs1 heterodimer and 25S rRNA on the 60S subunit. However, unlike L5, P34 and P37 may not interact with 25S rRNA.

Collectively, our analysis of the protein-RNA and protein-protein interactions within the 5S RNP has revealed several unique features of this complex in *T. brucei*. Our findings show that similar to what has been reported in fungi, the interactions between TbRpf2, 5S rRNA, and L5 exist; however, they interact in a unique manner in *T. brucei*. These studies also elucidate the role of RNAs in influencing the protein-protein interactions within the 5S RNP and also the order of assembly of the *T. brucei* 5S RNP. Previously our laboratory showed that 5S rRNA enhances the interaction between L5 and P34. Our studies here showed that 5S rRNA does not affect the interaction between TbRpf2 and L5 or between TbRpf2 and P34. These results support our hypothesis that 5S rRNA, L5, and P34 interact together first before interacting with Rpf2 during assembly.

All of the *in vitro* protein-RNA and protein-protein interactions have been characterized in the absence of Rrs1. These results suggest that the relationship between Rpf2 and Rrs1 is different in *T. brucei*. The structure of Rpf2-Rrs1 showed that Rpf2 is composed of two duplicated BRIX domains (Rpf2-N and Rpf2-C) and that Rrs1 completes the C-terminal BRIX domain of Rpf2 (12, 13, 21). These studies suggested that this domain duplication occurred in order to specify function where the N-terminal BRIX domain mediates RNA binding, while the C-terminal BRIX domain of Rpf2 mediates protein binding. The sequence alignment between TbRpf2 and ScRpf2 shows that there is a stretch of 18 residues present in the corresponding C-terminal BRIX domain of TbRpf2 that is absent in ScRpf2 (Fig. 2A). Furthermore, in the ScRpf2-Rrs1 structure, the region corresponding to these residues is partially disordered and located in the C-terminal BRIX domain of Rpf2 near where Rrs1 binds. On the basis of these differences, we hypothesize that there are unique interactions that take place in this region that are different between TbRpf2 and ScRpf2. These extra residues in TbRpf2 could provide binding sites for different factors or structurally shape the C-terminal domain of Rpf2 in a way that might influence binding interactions with Rrs1, 5S rRNA, or other binding factors.

We next characterized the function of Rpf2 in ribosome biogenesis in *T. brucei* using RNAi knockdown of the Rpf2 homolog. Upon induction of TbRpf2 RNAi, we observed that by day 3 postinduction cell growth was arrested and the cells began to die (Fig. 6A). The cells also exhibited defects in their ability to fully divide (Fig. 6B). We confirmed that the transcript and protein levels of TbRpf2 were reduced by day 2 postinduction (Fig. 6C and D). Collectively, these results show that TbRpf2 is an essential protein in *T. brucei*.

Using this RNAi cell line, we characterized the effect that loss of TbRpf2 has on the other members of the 5S RNP and also on ribosome biogenesis. We performed Western blot analysis of extracts prepared on days 0, 1, 2, and 3 postinduction and showed that knockdown of TbRpf2 led to a decrease in the protein expression levels of P34, P37, and L5 protein on days 2 and 3 postinduction. These results suggest that the absence of TbRpf2 is significant and leads to a defect in recruitment of P34, P37, and L5 to the 60S subunit. This suggests that the function of Rpf2 as an assembly factor is conserved in *T. brucei*.

We next characterized the effect of TbRpf2 knockdown on ribosome biogenesis. We observed that on day 2 postinduction, there is an accumulation of the 40S subunit, a decrease in the 60S subunit, a decrease in the formation of the 80S subunit, a decrease in polysome formation, and the formation of half-mers (Fig. 8B). This result suggests that loss of TbRpf2 leads to a defect in which functional 60S ribosomal subunits are not formed and are therefore unable to join with the 40S subunit. Thus, the 40S subunit accumulates and less 80S monosomes are formed. This phenotype has been observed in *S. cerevisiae* when Rpf2 was depleted (11). This suggests that the function of TbRpf2 in ribosome biogenesis could be similar to its described role in fungi. In order to further characterize these results and to determine whether in *T. brucei*, loss of Rpf2 leads to the formation of 60S subunits that are not competent for export, future studies will determine the cellular localization of the 60S preribosomal subunits when Rpf2 is knocked down.

In these studies, we have characterized several unique features of the TbRpf2 protein and its interaction with the 5S RNP in *T. brucei*. Similar to P34, P37, and L5 proteins, TbRpf2 is also an essential protein in *T. brucei* (17, 24). This supports the hypothesis that members of the 5S RNP are essential in other organisms beyond fungal model systems. The interaction of TbRpf2 with 5S rRNA and with L5 in *T. brucei* is also conserved. However, TbRpf2 also forms a novel interaction with trypanosome-specific proteins. Future work in our laboratory will focus on completing the characterization of the *T. brucei* 5S RNP components L11 and Rrs1. Additional studies will be performed to determine the order of assembly of the 5S RNP complex in *T. brucei*. Ribosomes are excellent targets for antibiotics due to their essential role in all organisms (25). By identifying the unique features involved in the assembly of the 5S RNP in *T. brucei*, we can potentially identify interactions involving trypanosome-specific factors that are suitable chemotherapeutic targets.

## MATERIALS AND METHODS

### Generation of PTP protein-tagged cell lines.

The entire coding sequences for P34, P37, and L5 were inserted into the pN-PURO-PTP plasmid (gift from Arthur Gunzl, University of Connecticut, Addgene plasmid no. 51711) to generate pN-PURO-PTP-P34, pN-PURO-PTP-P37, and pN-PURO-PTP-L5 plasmids (26). These plasmids were linearized using NotI and individually transfected into *Trypanosoma brucei* 427 procyclic cells to generate three stable cell lines expressing N-terminal protein A-tobacco etch virus (TEV)-protein C (PTP)-tagged proteins at an endogenous level. The cells were grown in Cunningham’s medium supplemented with 10% fetal bovine serum. Puromycin, at a final concentration of 4 μg/ml, was used to select for cells expressing the PTP-tagged proteins.

### PTP purification.

Transfected cells expressing PTP-L5, PTP-P34, and PTP-P37 were grown to a cell density of 2 × 10^7^ cells/ml and then sedimented at 4°C at 2,500 × *g* (Sorvall RC 5B plus, Sorvall SLA-300). The cell pellet was then washed once with transcription buffer (150 mM sucrose, 20 mM potassium l-glutamate, 3 mM magnesium chloride, 20 mM HEPES-KOH [pH 7.7], 2 mM dithiothreitol [DTT], and one tablet of protease inhibitor [Roche]) and homogenized. The lysate was used in tandem affinity purification as previously described (27). Purified PTP-tagged proteins and their associating proteins were separated by SDS-polyacrylamide gel electrophoresis, silver stained, and identified using mass spectrometry (Thermo Scientific LTQ Orbitrap Elite, Fred Hutchinson Cancer Research Center, Seattle, WA). The sorting and analysis were performed using Proteome Discoverer (Thermo Fisher Scientific) and Microsoft Excel.

### Recombinant proteins.

The entire coding sequences for TbRpf2, P34, and L5 proteins were individually cloned into the pTrcHis TOPO TA vector (Thermo Fisher Scientific) and expressed as N-terminal poly(His)_6_-tagged proteins in *Escherichia coli* TOP10 One Shot cells (Thermo Fisher Scientific). Expression and purification of recombinant proteins were performed as previously described (15). Eluted fractions of the recombinant proteins were analyzed by SDS-polyacrylamide gel electrophoresis and visualized by Coomassie blue staining and Western blotting. Fractions containing the recombinant proteins were pooled and frozen at −80°C as 250-μl fractions.

### *In vitro* transcription of 5S rRNA.

*T. brucei* 5S ribosomal DNA (rDNA) was cloned into pCR2.1 TOPO vector (Thermo Fisher Scientific) and PCR amplified as previously described (16). This template was then used for T3-driven *in vitro* transcription of radiolabeled [α-^32^P]UTP and unlabeled 5S rRNA (Ambion, Thermo Fisher Scientific). The transcribed 5S rRNA was separated from unincorporated nucleotides using Nuc-Away spin columns (Thermo Fisher Scientific). The transcribed RNA was heated at 55°C for 10 min and quickly cooled prior to each experiment.

### Filter binding assay.

Radiolabeled 5S rRNA was kept at a concentration always less than 0.5 nM and incubated with increasing concentrations (0 to 250 nM) of recombinant TbRpf2 protein. The protein-RNA mixture was incubated in binding buffer (10 mM Tris [pH 7.4], 1 mM EDTA, 100 mM NaCl, and 100 μg/ml bovine serum albumin [BSA]) for 30 min at room temperature. The filter binding experiment and the quantification of the binding affinity (*K*_*d*_) of 5S rRNA for the recombinant TbRpf2 protein were performed as previously described (28). The binding graph shows the average values for three biological replicates.

### Preparation of whole-cell extracts.

Cell extracts were prepared from a total of 1 × 10^8^ cells collected from *T. brucei* 427 procyclic cells. These cells were harvested at 6,000 × *g* (Eppendorf 5430R) for 10 min at 4°C and washed in SBG buffer (20 mM sodium phosphate [pH 7.9], 150 mM NaCl, and 20 mM glucose). Cell pellets were resuspended in DTE buffer (1 mM Tris [pH 8.0], 1 mM EDTA, and one tablet protease inhibitor [Roche]) and passed through a syringe 10 times using a 26-gauge needle. Extracts were then sedimented at 15,800 × *g* (Spectrafuge 24D) for 10 min at 4°C to isolate the supernatant (soluble protein).

### Immune capture.

Antibody-coated beads were prepared as previously described (29). The beads were coated with anti-Rpf2 antibody that was prepared by affinity purification of antibody raised against the TbRpf2 peptide ^122^GIIAADRMEMPRDE^135^ (Bethyl Laboratories). The antibody-coated beads were incubated with recombinant TbRpf2 plus L5 or P34 protein (1 μg each) for 1 h at 4°C with gentle rotation. P34 protein was used in these experiments, since P34 and P37 have been shown to be functionally interchangeable. After incubation, the supernatants were removed, and the beads, coated with antibody-protein complexes, were washed three times in phosphate-buffered saline with Tween 20 (PBS-T). The protein complexes were eluted from the beads by resuspending them in SDS sample buffer, followed by heating at 70°C for 10 min. The eluted fractions (bound proteins) together with the supernatant (unbound protein) and the input (antibody control, total protein in reaction) were analyzed by SDS-polyacrylamide gel electrophoresis, followed by Western blotting using either anti-L5 antibody (15) or anti-P34/P37 antibody (30). Whole-cell extracts (WCE) were treated with 10 μg/ml RNase A (Thermo Fisher Scientific) for 1 h at 37°C. We routinely perform self-blots as controls for our immune capture experiments, and we have provided an example in Fig. S3 in the supplemental material. The immune capture experiments were performed as three biological replicates, and representative blots are shown.

10.1128/mSphere.00394-17.3 FIG S3  Control experiment (self-blot) to detect TbRpf2 in the immune capture experiment. The proteins were incubated with beads cross-linked with anti-Rpf2 antibody to capture TbRpf2 (TbRpf2 migrates to 40 kDa). The immune capture (IC) was analyzed by Western blotting (Wb) using anti-Rpf2 antibody. No antibody was used for the Beads alone lanes. Lanes: I, input (Rpf2 antibody control, 50% [recombinant protein] of total protein used in the reaction); S, supernatant (unbound TbRpf2); P, pellet (bound TbRpf2); M, molecular mass markers (in kilodaltons). Download FIG S3, PDF file, 0.3 MB. Copyright © 2017 Kamina et al. 2017 Kamina et al. This content is distributed under the terms of the Creative Commons Attribution 4.0 International license.

### Generation of the Rpf2 knockdown cell line.

The tetracycline (Tet)-inducible Rpf2 RNAi plasmid p2T7-177-Rpf2 was constructed by insertion of a 560-nucleotide fragment (nucleotides 467 to 1026) from the coding region of Rpf2 (TriTrypDB accession no. Tb427.07.270) between two head-to-head T7 promoters under the control of tet operators. The p2T7-177-Rpf2 plasmid was linearized using NotI and transfected by electroporation (Amaxa Nucleofactor II) into procyclic 29-13 cells. This 29-13 strain, which expresses both T7 RNA polymerase and tetracycline repressor for Tet-regulated expression of genes, was grown as described above in media containing 15 μg/ml of G418 and 50 μg/ml of hygromycin to maintain the T7 RNA polymerase and tet repressor constructs. Cells expressing the p2T7-177-Rpf2 construct were selected with 2.5 μg/ml phleomycin, and clonal cell lines were generated through limiting dilutions. Expression of Rpf2 double-stranded RNA was induced with 2.5 μg/ml Tet. Growth curves for wild-type, uninduced, and tetracycline-induced TbRpf2 RNAi cells were generated to compare the effect of TbRpf2 knockdown on cell growth (based on cell density and total cell dilution). The growth curves were performed as three biological triplicates and the average values from these experiments are shown.

### Quantitative PCR (qPCR).

RNA was isolated from wild-type, uninduced, and Tet-induced Rpf2 RNAi cells using TRIzol reagent (Thermo Fisher Scientific). The isolated RNA was DNase I treated (Turbo DNA-free kit; Ambion) and then used to generate cDNA. The cDNA was prepared using random hexamers (Applied Biosystems) and SuperScript III first-strand synthesis supermix (Thermo Fisher Scientific). Real-time PCR was performed using IQ SYBR green supermix and primers shown in Table 1. The starting cDNA quantities were normalized to the quantity of telomerase reverse transcriptase (TERT). Reverse transcription-PCR (RT-PCR) was performed on three biological replicates, and the graph shows the average values from the three experiments.

**TABLE 1:** Oligonucleotides used in this study

Primer^a^	Sequence (5′-3′) (reference)
PTP-P34 F	GCG GCC GCA ATG GCC CCA AAG TCT
PTP-P34 R	CGA GCG GCC GCT CAC TGC TTC CTC TT
PTP-P37 F	GTA CGT AAG CGG CCG CAA TGG CCC CAA AGT CTG
PTP-P37 R	CAG TAT ACG GGC CCT TAC TTC CTC TTG GCA TCC T
PTP-L5 F	GAC GTA AGC GGC CGC AAT GAC ATT CG
PTP-L5 R	GCT ACG ATG GGC CCT TAC TTC GCA CG
P34 F	ATG GCC CCA AAG TCT GCT GC
P34 R	TCA CTG CTT CCT CTT GGC AT
Rpf2 F	ATG TCC TCT ACT GGT GGC TTC
Rpf2 R	TCA AAT ATC CCT ATC GGG GTT
Rpf2 RNAi F	TAG GGA TCC TGT CTG ACC CCA CAT TCG TA
Rpf2 RNAi R	CGA AAG CTT TTC TTC CCT CTG CTG CGT
Rpf2 FWD set 4	CGG TAG CAG TGA AAC GGA AAT A
Rpf2 REV set 4	CTT CCC AGT GCC ATA ACA ATC
TERT forv real-time	GAG CGT GTG ACT TCC GAA GG (32)
TERT rev real-time	AGG AAC TGT CAC GGA GTT TGC (32)
5ST3 Fwd	ATTAACCCTCACTAAAGGGTACGACCATACTTGGCC (16)
5S Rev	AGAGTACAACACCCCGGGT (16)

a Forward primers are indicated by a capital F at the end of the primer designation or by FWD, forv, or Fwd in the primer designation. Reverse primers are indicated by a capital R at the end of the primer designation or by REV, rev, or Rev in the primer designation.

### Western blot analysis.

Wild-type, uninduced, and TbRpf2 RNAi-induced cells were harvested and used for WCE preparation as described above. Proteins were separated by 4 to 12% SDS-polyacrylamide gel electrophoresis and then analyzed by Western blotting using antibodies directed against TbRpf2 (Bethyl Laboratory), L5 (15), and P34/P37 (30). HSP 70 was used as a loading control (31). The TbRpf2 antibody was used at a 1:500 dilution, the HSP 70 antibody was used at a 1:20,000 dilution, and all the other antibodies were used at a 1:1000 dilution. Li-Cor near-infrared fluorescent Western blotting was used for quantification. The signal obtained from the Western blot is directly proportional to the amount of target protein. All Western blot analyses were performed on three biological replicates, and representative blots are shown.

### Polysome profile.

Cell extracts were prepared from a total of 1 × 10^9^ cells collected from wild-type, uninduced, and RNAi-induced cells from day 2 postinduction. These extracts were prepared as previously described (17). Briefly, the cells were treated with cycloheximide (100 μg/ml) and then subjected to sedimentation on 10% to 40% sucrose gradients in order to separate the ribosomal subunits (40S and 60S), monosomes (80S), and polysomes. These samples were then analyzed by monitoring the UV absorbance at 260 nm. Experiments were performed in three biological replicates and a representative image is shown.
