# Supplementary material for: Essential Assembly Factor Rpf2 Forms Novel Interactions within the 5S RNP in Trypanosoma brucei
Source: mSphere. 2017 Oct 18;2(5):e00394-17. doi: 10.1128/mSphere.00394-17 (PMC5646243; doi:10.1128/mSphere.00394-17)
Supplement: TABLE S1 [file sph005172389st4.pdf]

Table S1: Proteins identified from PTP-L5 purification<br>60S ribosomal proteins				
**Protein Name (From Tb927 DB)**	**Peptides Identified**	**Unique peptides identified**	**Amino acid coverage (%)**	**Protein**
Tb927.9.5690	12	5	88.50%	60S acidic ribosomal protein
Tb927.7.1730	15	12	43.80%	L7
Tb927.4.2180	8	6	40.90%	L35A (L33 in yeast)
Tb927.3.5050	25	13	37.20%	L4
Tb927.11.15900	15	8	33.80%	L27
Tb927.10.3840	9	6	31.30%	L18a (L20 in yeast)
Tb927.3.3320	24	7	30.30%	L13
Tb927.9.11380	4	4	29.50%	L23
Tb927.10.11390	15	6	28.10%	L6
Tb927.7.5180	3	3	28.00%	L23a (L25)
Tb927.9.15210	5	3	27.50%	L36
Tb927.10.1100	7	5	26.50%	L9
Tb927.10.13500	10	7	26.30%	L10
Tb927.9.15170	27	10	26.30%	L5

Tb927.11.4820	12	5	25.30%	L17
Tb927.11.680	3	4	25.20%	L21E
Tb927.4.1790	10	8	23.30%	L3
Tb927.4.3550	8	5	20.30%	L13a (L16 in yeast)
Tb927.10.220	2	1	19.40%	L37a (l43 in yeast)
Tb927.9.12200	4	3	18.60%	L31
Tb927.9.5690	13	6	18.20%	60S acidic ribosomal subunit protein
Tb927.9.8420	7	5	17.80%	L10a (L1 in yeast)
Tb927.10.9800	3	2	17.70%	L22
Tb927.7.5020	10	5	16.90%	L19
Tb927.9.14370	9	2	16.50%	L26
Tb927.9.1850	3	3	14.70%	L35
Tb927.5.1110	8	2	14.20%	L2
Tb927.8.1330	3	4	13.50%	L7a (L8 in yeast)
Tb927.11.6200	5	5	13.00%	L28
Tb927.10.5460	3	2	13.00%	L24

Tb927.10.270	2	1	11.20%	L32
Tb927.9.7590	2	1	9.10%	L11

**Table S1 continued: Proteins identified from PTP-L5 purification 40S ribosomal proteins**				
**Protein Name (From Tb927 DB)**	**Peptides Identified**	**Unique peptides identified**	**Amino acid coverage (%)**	**Protein**
Tb927.11.3600	21	13	40.70%	S4
Tb927.10.11540	11	7	37.90%	S3
Tb927.10.3940	14	9	37.50%	S3a (S1 in yeast)
Tb927.9.3990	13	9	34.70%	S7
Tb927.7.1040	7	5	30.90%	S16
Tb927.10.5340	11	7	30.70%	S18
Tb927.10.8430	10	4	28.90%	S12
Tb927.10.190	18	8	27.60%	S6
Tb927.10.3940	5	4	26.80%	S17
Tb927.7.240	5	3	25.20%	S33 (S28 in yeast)
Tb927.10.5610	9	5	24.20%	S9
	4	3	23.80%	

Tb927.10.1080				S23
Tb927.10.560	12	4	22.40%	S11
Tb927.10.7330	4	2	20.40%	S24E
Tb927.10.5370	4	3	18.00%	S10
Tb927.6.4980	9	3	17.40%	S14
Tb927.8.6150	7	3	15.90%	S8
Tb927.11.6300	3	3	13.70%	S5
Tb927.11.10790	6	3	13.50%	SA (S0 in yeast)
Tb927.11.6510	2	2	12.90%	S21
Tb927.2.5910	3	2	11.90%	S13

Table S1 continued: Proteins Identified from PTP-L5 purification non-ribosomal proteins					
**Protein Name (From Tb927 DB)**	**Peptides Identified**	**Unique peptides identified**	**Amino acid coverage (%)**	**Protein**	**Comments**
Tb927.9.12890	4	3	25.00%	Hypothetical protein	
Tb927.7.270	15	9	22.70%	Ribosome biogenesis protein	Rpf2
Tb927.11.14000	5	6	17.90%	TbP34/P37	
Tb927.10.5300	2	2	12.50%	Eukaryotic translation initiation factor 6 (eIF-6)	
Tb927.2.4710	5	4	11.10%	RNA binding protein	
Tb927.10.14680	3	3	9.40%	Ribosome biogenesis protein	BRX1
Tb927.11.3120	7	5	8.40%	NOG1	
Tb927.7.7050	11	6	8.10%	Hypothetical protein	
Tb927.11.4190	3	3	5.80%	Hypothetical protein	
