# Supplementary material for: Essential Assembly Factor Rpf2 Forms Novel Interactions within the 5S RNP in Trypanosoma brucei
Source: mSphere. 2017 Oct 18;2(5):e00394-17. doi: 10.1128/mSphere.00394-17 (PMC5646243; doi:10.1128/mSphere.00394-17)
Supplement: TABLE S2 [file sph005172389st5.pdf]

| Table S2: Proteins identified from PTP-P34 purification<br>60S ribosomal proteins | | | | |
|---|---|---|---|---|
| **Protein Name (From Tb927 DB)** | **Peptides Identified** | **Unique peptides identified** | **Amino acid coverage (%)** | **Protein** |
| Tb927.9.5690 | 11 | 5 | 88.50% | 60S acidic ribosomal protein |
| Tb927.10.1100 | 21 | 9 | 47.10% | L9 |
| Tb927.7.5180 | 15 | 10 | 43.90% | L23a (L25) |
| Tb927.4.2180 | 12 | 8 | 41.60% | L35a (L33 in yeast) |
| Tb927.7.1730 | 24 | 12 | 40.10% | L7 |
| Tb927.10.11390 | 16 | 8 | 39.10% | L6 |
| Tb927.10.3840 | 12 | 8 | 39.10% | L18a (L20 in yeast) |
| Tb927.3.5050 | 29 | 16 | 35.60% | L4 |
| Tb927.3.5050 | 10 | 7 | 35.30% | L23 |
| Tb927.10.13500 | 25 | 11 | 34.30% | L10 |

| | | | | |
|---|---|---|---|---|
| Tb927.3.3320 | 32 | 8 | 33.00% | L13 |
| Tb927.11.15900 | 17 | 7 | 32.30% | L27 |
| Tb927.11.680 | 8 | 5 | 32.10% | L21E |
| Tb927.9.15170 | 21 | 10 | 31.20% | L5 |
| Tb927.6.5120 | 3 | 3 | 30.80% | 60 acidic ribosomal protein P2 |
| Tb927.11.4820 | 20 | 6 | 27.70% | L17 |
| Tb927.9.15210 | 7 | 3 | 27.50% | L36 |
| Tb927.8.1330 | 10 | 7 | 24.60% | L7a (L8 in yeast) |
| Tb927.4.1790 | 20 | 12 | 23.50% | L3 |
| Tb927.10.270 | 2 | 2 | 9.85% | L32 |
| Tb927.4.3550 | 15 | 6 | 22.50% | L13a (L16 in yeast) |

| | | | | |
|---|---|---|---|---|
| Tb927.9.14370 | 5 | 3 | 22.40% | L26 |
| Tb927.11.6200 | 4 | 4 | 21.20% | L28 |
| Tb927.10.3380 | 4 | 2 | 21.10% | 60S acidic ribosomal protein P2 |
| Tb927.5.1110 | 11 | 6 | 20.80% | L2 |
| Tb927.9.1850 | 10 | 3 | 19.70% | L35 |
| Tb927.10.220 | 5 | 1 | 19.40% | L37a (L43 in yeast) |
| Tb927.9.12200 | 5 | 4 | 19.10% | L31 |
| Tb927.9.5690 | 21 | 6 | 18.20% | 60S acidic ribosomal subunit protein |
| Tb927.9.8420 | 6 | 4 | 17.80% | L10a (L1 in yeast) |
| Tb927.10.9800 | 4 | 2 | 17.70% | L22 |
| Tb927.7.5020 | 10 | 5 | 16.90% | L19 |

| | | | | |
|---|---|---|---|---|
| Tb927.8.6030 | 12 | 2 | 16.50% | L12 |
| Tb927.10.12680 | 6 | 2 | 14.10% | L34 |
| Tb927.10.5460 | 5 | 3 | 12.20% | L24 |
| Tb927.6.5040 | 4 | 1 | 11.20% | L15 |
| Tb927.11.4300 | 4 | 2 | 10.80% | L18 |
| Tb927.9.7590 | 2 | 2 | 7.20% | L11 |
| | | | | |

| **Table S2 continued: Proteins identified from PTP-P34 purification<br>40S ribosomal proteins** | | | | |
|---|---|---|---|---|
| **Protein Name (From Tb927 DB)** | **Peptides Identified** | **Unique peptides identified** | **Amino acid coverage (%)** | **Protein** |
| Tb927.11.3600 | 84 | 23 | 61.90% | S4 |

| | | | | |
|---|---|---|---|---|
| Tb927.10.3940 | 54 | 15 | 52.00% | S3a (S1 in yeast) |
| Tb927.11.3600 | 17 | 12 | 50.00% | S17 |
| Tb927.7.1040 | 27 | 11 | 47.70% | S16 |
| Tb927.10.11540 | 36 | 8 | 41.60% | S3 |
| Tb927.10.5340 | 25 | 10 | 40.50% | S18 |
| Tb927.4.1860 | 16 | 7 | 38.90% | S19 |
| Tb927.9.3990 | 36 | 12 | 38.60% | S7 |
| Tb927.2.5910 | 5 | 5 | 37.10% | S13 |
| Tb927.10.14710 | 19 | 8 | 36.80% | S2 |
| Tb927.10.190 | 35 | 12 | 34.00% | S6 |
| Tb927.6.4980 | 30 | 6 | 34.00% | S14 |
| Tb927.10.1080 | 11 | 4 | 29.40% | S23 |
| Tb927.10.8020 | 19 | 4 | 28.90% | S12 |
| Tb927.10.5610 | 17 | 6 | 28.40% | |

| | | | | |
|---|---|---|---|---|
| | | | | S9 |
| Tb927.8.6150 | 13 | 5 | 28.20% | S8 |
| Tb927.10.560 | 19 | 6 | 27.00% | S11 |
| Tb927.10.5370 | 12 | 4 | 26.20% | S10 |
| Tb927.10.2840 | 6 | 3 | 25.70% | S25 |
| Tb927.10.7330 | 13 | 3 | 25.50% | S24E |
| Tb927.7.240 | 11 | 3 | 25.20% | S33 ( S28 in yeast) |
| Tb927.11.6300 | 12 | 7 | 24.70% | S5 |
| Tb927.11.8200 | 5 | 2 | 22.50% | S26 |
| Tb927.10.5030 | 3 | 3 | 21.90% | S27a (S31 in yeast) |
| Tb927.11.6140 | 11 | 3 | 18.50% | S15a (S22 in yeast) |
| Tb927.7.2340 | 15 | 2 | 17.80% | S15 |
| Tb927.11.10790 | 26 | 4 | 13.90% | SA (S0 in yeast) |

| | | | | |
|---|---|---|---|---|
| Tb927.11.6510 | 8 | 2 | 12.90% | S21 |

| **Table S2 continued: Proteins identified from PTP-P34 purification<br>Non-ribosomal proteins** | | | | | |
|---|---|---|---|---|---|
| **Protein Name (From Tb927 DB)** | **Peptides Identified** | **Unique peptides identified** | **Amino acid coverage (%)** | **Protein** | **Comments** |
| Tb927.11.14020 | 24 | 11 | 40.10% | TbP34/P37 | |
| Tb927.6.1470 | 7 | 7 | 22.00% | Hypothetical protein | |
| Tb927.2.4710 | 7 | 7 | 19.00% | RNA binding protein | Contains RRM domains and Zf-CCHC |
| Tb927.9.6870 | 3 | 2 | 14.20% | RNA binding protein | Ribose operon repressor |
| Tb927.10.14680 | 6 | 4 | 14.00% | Ribosome biogenesis protein | BRX1 |

| | | | | | |
|---|---|---|---|---|---|
| Tb927.11.16400 | 2 | 2 | 12.00% | Kinetoplast-DNA associated protein | |
| Tb927.10.13140 | 2 | 2 | 11.60% | Hypothetical protein | |
| Tb927.8.2330 | 3 | 2 | 11.10% | Hypothetical protein | |
| Tb927.11.3120 | 5 | 5 | 8.40% | NOG1 (nucleolar GTP-binding protein 1) | |
| Tb927.3.5400 | 4 | 4 | 8.00% | Hypothetical protein | |
| Tb927.7.700 | 3 | 3 | 5.60% | Hypothetical protein | Nucleolar protein 10 |
| Tb927.11.4190 | 2 | 2 | 3.70% | Hypothetical protein | |
| Tb927.9.8820 | 2 | 2 | 2.60% | Hypothetical protein | |
