# Supplementary material for: Essential Assembly Factor Rpf2 Forms Novel Interactions within the 5S RNP in Trypanosoma brucei
Source: mSphere. 2017 Oct 18;2(5):e00394-17. doi: 10.1128/mSphere.00394-17 (PMC5646243; doi:10.1128/mSphere.00394-17)
Supplement: TABLE S3 [file sph005172389st6.pdf]

Table S3: Proteins identified from PTP-P37 purification<br>60S ribosomal proteins				
Protein Name<br>(From Tb927 DB)	Peptides Identified	Unique peptides identified	Amino acid coverage (%)	Protein
Tb927.9.5690	15	5	73.50%	60S acidic ribosomal protein
Tb927.10.1100	21	10	46.00%	L9
Tb927.7.1730	25	14	44.60%	L7
Tb927.4.2180	16	9	43.00%	L35A (L33 in yeast)
Tb927.11.680	12	7	41.50%	L21E
Tb927.10.3840	22	8	39.10%	L18a (L20 in yeast)
Tb927.10.11390	18	9	39.10%	L6
Tb927.10.13500	23	11	38.00%	L10
Tb927.9.14370	7	4	35.70%	L26
Tb927.7.5180	14	8	34.80%	L23a (L25)
Tb927.3.5050	45	14	34.50%	L4

Tb927.3.5050	13	6	34.50%	L23
Tb927.11.15900	15	8	33.80%	L27
Tb927.3.3320	38	9	33.00%	L13
Tb927.9.15170	29	11	31.20%	L5
Tb927.6.5120	4	3	30.80%	60S acidic ribosomal protein, P2
Tb927.9.5690	27	6	29.30%	60S acidic ribosomal subunit protein
Tb927.11.6200	6	5	28.80%	L28
Tb927.11.3230	5	4	28.30%	L44
Tb927.8.1330	9	8	27.50%	L7a (L8 in yeast)
Tb927.9.15210	8	3	27.50%	L36

Tb927.9.12200	6	5	25.50%	L31
Tb927.11.4820	21	5	25.30%	L17
Tb927.4.1790	40	11	24.90%	L3
Tb927.10.3380	6	3	21.90%	60S acidic ribosomal protein, P2
Tb927.5.1110	11	7	20.80%	L2
Tb927.11.10160	4	3	20.80%	L22
Tb927.7.5020	12	6	20.00%	L19
Tb927.4.3550	22	5	19.40%	L13a (L16 in yeast)
Tb927.10.220	9	1	19.40%	L37a (L43 in yeast)
Tb927.8.6030,	13	3	18.30%	L12
Tb927.9.8420	11	6	17.80%	L10a (L1 in yeast)
Tb927.9.7590	5	4	16.50%	L11

Tb927.10.270	2	3	15.90%	L32
Tb927.11.9730	7	3	15.30%	L34
Tb927.10.3280	3	2	13.40%	L38
Tb927.10.5460	6	2	10.80%	L24
Tb927.6.5040	4	1	9.50%	L15
Tb927.10.9880	3	2	9.30%	L30

**Table S3 continued: Proteins identified from PTP-P37 purification**<br>**40S ribosomal proteins**				
**Protein Name**<br>**(From Tb927 DB)**	**Peptides Identified**	**Unique peptides identified**	**Amino acid coverage (%)**	**Protein**
Tb927.11.3600	86	23	60.80%	S4
Tb927.10.3940	61	15	49.20%	S3a (S1in yeast)
	24	9	46.50%	

Tb927.11.3600				S17
Tb927.7.1040	27	8	43.00%	S16
Tb927.10.5340	32	9	40.50%	S18
Tb927.4.1860	43	8	40.10%	S19
Tb927.9.8820	68	12	38.60%	S7
Tb927.10.190	76	16	38.00%	S6
Tb927.10.14710	35	10	37.20%	S2
Tb927.6.4980	38	8	35.40%	S14
Tb927.10.8430	19	5	33.80%	S12
Tb927.11.9720	14	3	32.60%	S27
Tb927.2.5910	11	7	32.50%	S13
Tb927.10.5370	24	6	30.80%	S10
Tb927.10.11540	34	7	30.40%	S3
Tb927.11.10790	49	7	30.30%	SA (S0 in yeast)

Tb927.8.6150	10	5	30.00%	S8
Tb927.11.8200	5	3	29.70%	S26
Tb927.10.5610	33	7	29.50%	S9
Tb927.10.1080	20	4	29.40%	S23
Tb927.11.6140	11	4	29.20%	S15a (S22 in yeast)
Tb927.10.2840	11	6	28.30%	S25
Tb927.10.560	17	6	27.00%	S11
Tb927.10.7330	24	4	25.50%	S24E
Tb927.7.240	8	3	25.20%	S33 (S28 in yeast)
Tb927.7.240	8	7	24.70%	S5
Tb927.7.2340	6	2	17.80%	S15
Tb927.11.6510	7	2	12.90%	S21
Tb927.10.5030	10	2	8.60%	S27a (S31 in yeast)

Table S3 continued: Proteins identified from PTP-P37 purification<br>Non-ribosomal proteins					
**Protein Name (From Tb927 DB)**	**Peptides Identified**	**Unique peptides identified**	**Amino acid coverage (%)**	**Protein**	**Comments**
Tb927.11.14020	34	13	43.70%	TbP34/P37	
Tb927.2.4710	21	10	28.30%	RNA-binding protein	Part of RRM superfamily and contains two Zf-CC
Tb927.11.6320	15	8	28.20%	Hypothetical protein	MRB1 (mitochondrial RNA binding complex 1)
Tb927.6.1470	13	10	26.00%	Hypothetical protein	
Tb927.3.5400	14	12	25.00%	Hypothetical protein	
Tb927.10.14680	9	7	23.50%	Ribosome biogenesis protein	BRX1
Tb927.10.14700	24	7	22.80%	Hypothetical protein	
Tb927.10.2240	23	14	21.10%	Hypothetical protein	

Tb927.9.10770	11	8	18.20%	Poly (A)-binding protein 1	
Tb927.4.3060	4	3	16.90%	Hypothetical protein	
Tb927.9.11840	4	4	16.00%	Hypothetical protein	
Tb927.11.10860	5	5	15.50%	Hypothetical protein	
Tb927.9.15060	4	4	15.10%	rRNA processing protein	
Tb927.11.3120	9	9	14.40%	NOG1 (nucleolar GTP-binding protein 1)	
Tb972.11.9200	7	5	14.30%	Hypothetical protein	
Tb927.9.6870	2	2	14.20%	RNA-binding protein	Ribose operon repressor
Tb927.7.700	8	6	13.10%	Hypothetical protein	Nucleolar protein 10
Tb927.1.1700	4	6	13.00%	Putative uncharacterized protein	
Tb927.10.13140	4	3	12.20%	Hypothetical protein	
Tb927.8.2330	3	2	11.10%	Hypothetical protein	
Tb927.11.4190	7	5	10.80%	Hypothetical protein	

Tb927.6.2840	4	4	10.50%	Hypothetical protein	
Tb927.9.8820	12	8	10.00%	Hypothetical protein	
Tb927.3.3950	6	4	9.30%	Hypothetical protein	
Tb927.3.2750	7	5	8.60%	Hypothetical protein	
Tb927.9.13350	4	3	8.00%	Hypothetical protein	
Tb927.6.3690	2	2	7.30%	Pre-mRNA cleavage complex II Clp1-like	
Tb927.7.270	3	2	6.60%	Ribosome biogenesis protein	Rpf2
Tb927.10.14820	2	2	6.20%	Mitochondrial carrier protein	
Tb927.10.15360	4	4	2.70%	Unknown protein	
