# Supplementary material for: Essential Assembly Factor Rpf2 Forms Novel Interactions within the 5S RNP in Trypanosoma brucei
Source: mSphere. 2017 Oct 18;2(5):e00394-17. doi: 10.1128/mSphere.00394-17 (PMC5646243; doi:10.1128/mSphere.00394-17)
Supplement: FIG S1 [file sph005172389sf1.pdf]

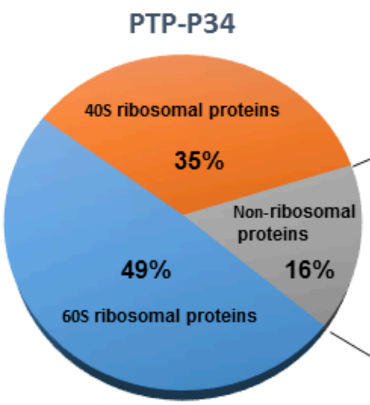

Proteins identified from PTP-P34 purification non-ribosomal proteins					
Protein Name (From Tb927 DB)	Peptides Identified	Unique peptides identified	Amino acid coverage (%)	Protein	Comments
Tb927.11.14020	24	11	40.10%	TbP34/P37	
Tb927.6.1470	7	7	22.00%	Hypothetical protein	
Tb927.2.4710	7	7	19.00%	RNA binding protein	Part of RRM superfamily and contains two Zf-CCHC
Tb927.9.6870	3	2	14.20%	RNA binding protein	Ribose operon repressor
Tb927.10.14680	6	4	14.00%	Ribosome biogenesis protein	BRX1
Tb927.8.2330	3	2	11.10%	Hypothetical protein	
Tb927.11.3120	5	5	8.40%	NOG1 (nucleolar GTP-binding protein 1)	
Tb927.3.5400	4	4	8.00%	Hypothetical protein	
Tb927.7.700	3	3	5.60%	Hypothetical protein	Nucleolar protein 10
