# Supplementary material for: Essential Assembly Factor Rpf2 Forms Novel Interactions within the 5S RNP in Trypanosoma brucei
Source: mSphere. 2017 Oct 18;2(5):e00394-17. doi: 10.1128/mSphere.00394-17 (PMC5646243; doi:10.1128/mSphere.00394-17)
Supplement: FIG S2 [file sph005172389sf2.pdf]

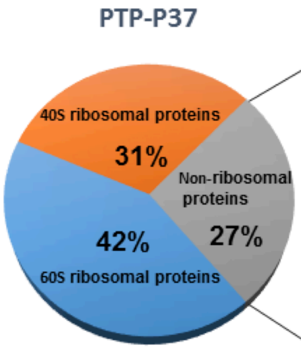

Proteins identified from PTP-P37 purification non-ribosomal proteins					
Protein Name (From Tb927 DB)	Peptides Identified	Unique peptides identified	Amino acid coverage (%)	Protein	Comments
Tb927.11.14020	34	13	43.70%	TbP34/P37	
Tb927.2.4710	21	10	28.30%	RNA-binding protein	Part of RRM superfamily and contains two Zf-CCHC
Tb927.11.6320	15	8	28.20%	Hypothetical protein	MRB1 (mitochondrial RNA binding complex 1)
Tb927.10.14680	9	7	23.50%	Ribosome biogenesis protein	BRX1
Tb927.9.10770	11	8	18.20%	Poly (A)-binding protein 1	
Tb927.9.15060	4	4	15.10%	rRNA processing protein	
Tb927.11.3120	9	9	14.40%	NOG1 (nucleolar GTP-binding protein 1)	
Tb927.7.700	8	6	13.10%	Hypothetical protein	Nucleolar protein 10
Tb927.7.270	3	2	6.60%	Ribosome biogenesis protein	Rpf2
