# Supplementary figures and images for: Essential Assembly Factor Rpf2 Forms Novel Interactions within the 5S RNP in Trypanosoma brucei
Source: mSphere. 2017 Oct 18;2(5):e00394-17. doi: 10.1128/mSphere.00394-17 (PMC5646243; doi:10.1128/mSphere.00394-17)

IC: anti-Rpf2

Wb: anti-Rpf2

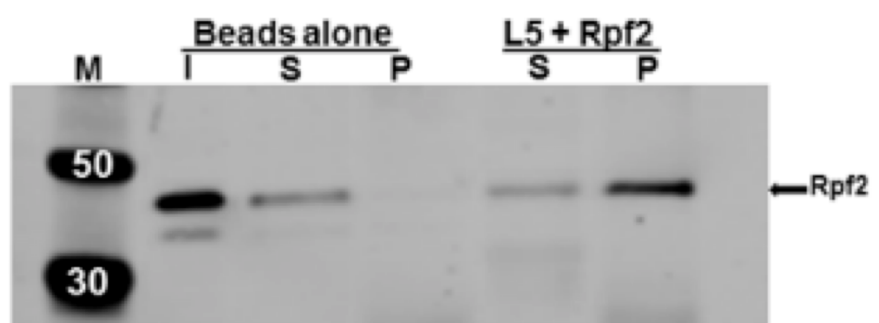

Supplement: FIG S3 [file sph005172389sf3.pdf]
